# Development of a Cloud Computing-Based Pier Type Port Structure Stability Evaluation Platform Using Fiber Bragg Grating Sensors

**DOI:** 10.3390/s18061681

**Published:** 2018-05-23

**Authors:** Byung Wan Jo, Jun Ho Jo, Rana Muhammad Asad Khan, Jung Hoon Kim, Yun Sung Lee

**Affiliations:** Department of Civil and Environmental Engineering, Hanyang University, Seoul 04763, Korea; joycon@hanmail.net (B.W.J.); masadkhan87@gmail.com (R.M.A.K.); junghoon3301@hotmail.com (J.H.K.); nikeshoo@nate.com (Y.S.L.)

**Keywords:** port structure, Fiber Bragg Grating Sensor, cloud computing, structure stability evaluation, structure monitoring

## Abstract

Structure Health Monitoring is a topic of great interest in port structures due to the ageing of structures and the limitations of evaluating structures. This paper presents a cloud computing-based stability evaluation platform for a pier type port structure using Fiber Bragg Grating (FBG) sensors in a system consisting of a FBG strain sensor, FBG displacement gauge, FBG angle meter, gateway, and cloud computing-based web server. The sensors were installed on core components of the structure and measurements were taken to evaluate the structures. The measurement values were transmitted to the web server via the gateway to analyze and visualize them. All data were analyzed and visualized in the web server to evaluate the structure based on the safety evaluation index (SEI). The stability evaluation platform for pier type port structures involves the efficient monitoring of the structures which can be carried out easily anytime and anywhere by converging new technologies such as cloud computing and FBG sensors. In addition, the platform has been successfully implemented at “Maryang Harbor” situated in Maryang-Meyon of Korea to test its durability.

## 1. Introduction

More than 25% of the port facilities in Korea were developed in the 1960s and 1970s. With the passage of time, these facilities have deteriorated and, most of them have already deteriorated. For instance, Breakwater was installed on Gageo Island over 30 years ago and, since its construction, it has faced many instances of severe conditions, including typhoon Muifa in August 2011. Thus, it is important to maintain port structures and constantly monitor these structures. Moreover, in developed countries such as Italy, it is common to dedicate more than 30% of the national civil work expenditure for the maintenance of structures. On the other hand, in the case of Korea, the share for the maintenance cost is less than 10% of the total construction cost of port structures. The maintenance cost is most likely expected to increase with the aging of port structures. Thus, inspection technology is becoming an issue of national importance to maintain port facilities [1,2].

The existing port facility maintenance management system performs a safety evaluation of port facilities by performing inspections and diagnosis according to the “Special Act on Facility Management”, but most of the inspection and diagnostic survey methods are based on visual inspections [3,4]. In land inspections, it is easy to follow the status of inspections which are carried out by the naked eye, and the accuracy of the diagnosis is maximized due to the advancement of inspection equipment and surveying equipment. Underwater inspections are also accompanied by visual inspection and wave irradiation using divers. Usually, it is difficult to accurately investigate the port structure as shellfish and pollutants are attached to the surface of underwater structures, which require cleaning before examination in addition to many other preparations. Visual inspections are not accurate and have many disadvantages, including the subjective opinion of investigators [5,6]. Moreover, to address the latest demands of objective data collection for the evaluation of structures, it is necessary to use sophisticated instruments for the monitoring of such structures.

In addition, most domestic underwater diagnoses have been made using conventional methods using divers. To prevent decompression sickness, it is necessary to perform diving for only two hours at a water depth of 20 m followed by rest for six hours in an eight-hour shift. Due to the limited number of working hours, it is difficult to make precise diagnosis and the work efficiency is significantly reduced when marine conditions are poor. Moreover, the supervisor does not check the investigation process directly but relies only on the diver, which leads to the possibility of misdiagnosis. Therefore, new inspection technologies are required due to the working time limit and inspection quality issues [3,4].

Structure maintenance management systems have become more sophisticated and accurate as a result of technological advancements such as the Fiber Bragg Grating (FBG) sensor. The FBG sensor refers to a fiber optic device fabricated by employing the phenomenon in which the refractive index is increased by exposing a portion of the optical fiber core doped with germanium (GE) to a strong ultraviolet ray based on Bragg’s law [7,8]. The sensor detects variations of the properties of light travelling through the optical fiber. Therefore, an FBG sensor can be installed in various structures to detect multiple physical transformations using an automatic measurement system. The transformation detected by the sensor can be converted into numerical values or graphs to efficiently evaluate the safety of the structure [9,10]. The advantages of the sensor include its flexibility, high sustainability, long-term stability, high durability, and high immunity to electrical or magnetic interference, which are key to structural health monitoring [5,11,12,13].

Furthermore, rapid growth of new technologies such as Internet of Things, wireless sensor network, and cloud computing has demonstrated the new capabilities and methods for a structural health monitoring in many researches. Structural health monitoring combined with automatic data processing for stability evaluation can be a promising direction for enhancing the structural safety of harbor structures [14,15,16,17,18]. Specifically, automatic data processing offers unique advantages in its use with harbor structure, which is difficult to monitor considering its size and depth. The monitoring will have great potential of minimizing the deterioration of the structures and preventing any accidents from the damages.

Among the many types of harbor structures, pier type structures form a complicated structure system as a pier-type erection facility with several steel pipe piles which make it difficult to monitor and evaluate the condition of the structure. Therefore, in this research, a cloud computing-based pier type harbor structure stability evaluation platform using FBG sensors was developed for efficient maintenance and stability enhancement of structures. In addition, the server of the platform will automatically alert a manager via a smartphone when the safety evaluation index is low to prevent accidents. The platform, which combines the safety evaluation system of the port structures with the FBG sensors and a cloud computing, will be an integrated facility management system which can be monitored continuously as part of the regular inspection of structures. Although port structures are wide and deep, which make it difficult to observe and evaluate the structure, port structures can be continuously monitored at anywhere by converging cloud computing platforms to structural health monitoring. Furthermore, it will be possible to minimize casualties and economic damage caused by the sudden destruction of the facilities by monitoring the structure and employing a rapid alert system.

## 2. Sensor Selection

The FBG sensors, gateway, and cloud computing-based web server were installed as part of the cloud computing-based pier type harbor structure stability evaluation platform to monitor and evaluate structures. In this study, three types of FBG sensors were selected for monitoring the ocean environment: FBG strain sensor, FBG displacement gauge, and FBG angle meter. The gateway connects the FBG sensors with the web server which gathers data from the sensors and transmits the data to the server. Furthermore, Amazon Web Services was used as the web server of the platform to analyze, visualize, save, and execute the data collected from the FBG sensors.

### 2.1. FBG Sensors

**Figure 1:**
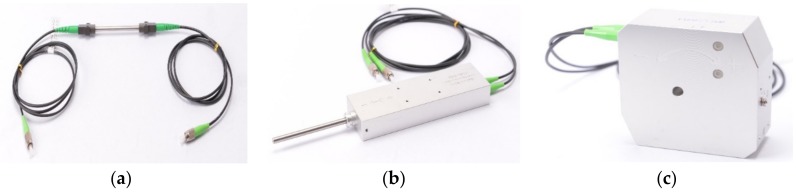
(**a**) FBG strain sensor; (**b**) FBG displacement gauge; and (**c**) FBG angle meter.

An FBG is a periodic structure in which a reflector is fabricated in a short segment of the core of an optical fiber by revealing the fiber to an intense UV light. The calculation method utilizes the change of the refractive index inside the sensor when the variation is applied. However, FBG sensors are very sensitive and they must be installed bare in a structure where the harsh environment of a port structure affects the durability of sensors [7,8]. Therefore, the FBG strain sensor, FBG displacement gauge, and FBG angle meter were used, as shown in Figure 1 and tested for the ocean environment.

The FBG sensors are sensitive to external temperature change which has a strong influence on shift change in wavelength. When FBG sensors are installed in the marine environment where the temperature changes significantly over time, the accuracy and performance of the sensors can be unintentionally affected. To eliminate the inadvertent effect of temperature change on strain, displacement, and angle measurements, the FBG sensors are designed to measure the wavelength of the temperature change and automatically reflect it to the measurements. Therefore, the temperature-induced shift wavelength can be expressed as the following Equation (1):

(1) $$\frac{\Delta \lambda_{F}}{\lambda_{F}}=\left(1-P_{p}\right)\Delta \varepsilon +\left[\frac{1}{n}{\zeta +\alpha }_{t}\right]\Delta T$$

where ∆λ_F_/λ_F_ is the ratio of the displacement wavelength to the Bragg’s wavelength, and n is the reflective index of the fiber core. In addition, P_p_, ζ and α_t_ are the coefficient for photoelastic, thermos-optical, and thermal expansion, respectively. The wavelength varies with changes in temperature increment, ∆T, and the strain increment, ∆ε [7].

**Figure 2:**
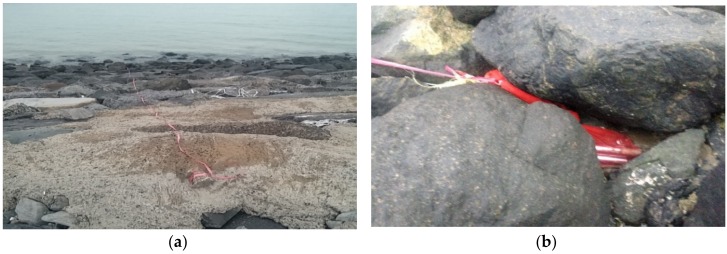
Sea water test of the materials for the cover of the FBG sensors: (**a**) during the tide; and (**b**) during the ebb tide.

In addition, a protective cover for the FBG sensors was installed and tested in the marine environment to protect the sensors from corrosions or long-term attachment of barnacles and floats. To select the most suitable material for the cover, three types of materials were used for functional test: anodized aluminum, stainless steel (SUS 304), and stainless steel (SUS316L). The materials were repeatedly submerged under the ocean during the tide and exposed in the air during the ebb tide twice a day to select the materials for the covers of the FBG sensors as shown in Figure 2.

**Figure 3:**
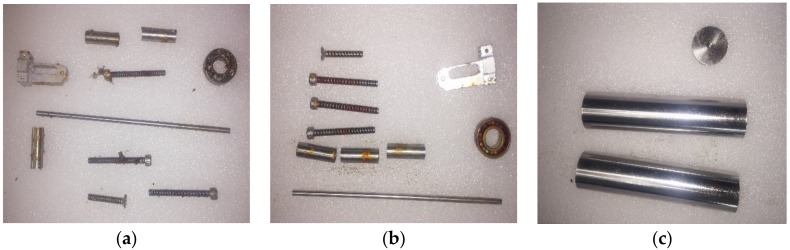
Result for the sea water test of: (**a**) an anodized aluminum cover; (**b**) a stainless steel (SUS304); and (**c**) a stainless steel (SUS316L).

As Figure 3 demonstrates, a stainless-steel cover (SUS 304) and an anodized aluminum cover began to rust from corners of bolts after one month and two months, respectively. However, a stainless-steel (SUS316L) did not show any signs of corrosion for a year. Based on the functional test result, a stainless steel cover (SUS316L) is selected as the most suitable for covering the FBG sensors.

#### 2.1.1. Selection of the FBG Strain Sensor

Most conventional FBG strain sensors are made of integral molding in which simultaneous deformation of the FBG and packaging degrades the measurement accuracy. Therefore, FBG strain sensors were developed in such a way that selective separation of the sensor and protective package is possible, thus minimizing strain deformation caused by temperature. In addition, the sensors are easy to install in any shape or type of structure such as a concrete surface, making them suitable for various applications. The measurement range for the FBG strain sensor is ±2000 με, where the sensitivity of the sensor is greater than or equal to 1 pm/με. However, the sensor can be installed inside and outside of the structure such that it is possible to constantly and precisely measure long-term strain. In addition, the operation temperature range is wide such that the gauge enables precise and stable measurements in various environments. Table 1 summarizes the specifications of the FBG strain sensor.

#### 2.1.2. Selection of the FBG Displacement Gauge

The FBG displacement gauge was developed in a similar manner as the FBG strain sensor, allowing selective separation of the package and sensor. The differences between the FBG displacement gauge and FBG strain sensor are reflected in their specifications, as shown in Table 2. The measurement range of the sensor was developed to be less than or equal to 30 mm and the sensitivity of the sensor was designed to be greater than or equal to 100 pm/mm. The main specifications of the FBG displacement gauge are illustrated in Table 2.

#### 2.1.3. Design of the FBG Angle Meter

The principle of the FBG angle meter is that it directly measures the angle change of the structure based on the wavelength change due to the movement of a weight connected directly to the fiber optic sensor. However, a measurement range of 6° with a sensitivity of greater than or equal to 450 pm/° are the main specifications for the developed FBG angle meter, as shown in Table 3. In addition, the meter is waterproofed by using an O ring and waterproof epoxy in the connection of the angle meter and the package, which is graded IP68.

#### 2.1.4. Selection of the Cable for Sea Water

**Figure 4:**
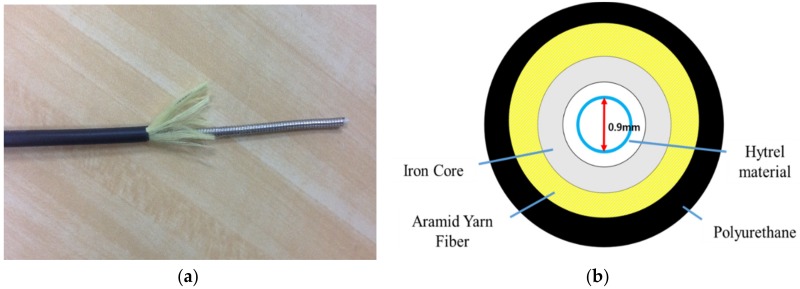
(**a**) Developed cable of the FBG sensors for sea water; and (**b**) cross-section of the cable.

Since the FBG sensors are installed in the ocean environment, the sensors’ cables are protected as Figure 4 describes. The 0.9 mm hytrel material is used for the fiber optic sensor protection tube. In addition, an iron core and aramid yarn fiber protective cloth are used to protect the fiber optic sensor from compressive and tensile forces. Polyurethane is used for the covering which is resistant to flames and has excellent durability against external environments such as seawater. Therefore, the durability of the cable is strong against sea water, chemicals, and organic solvents because of the multiple coverings.

#### 2.1.5. Submergence Test of the FBG Sensors

**Figure 5:**
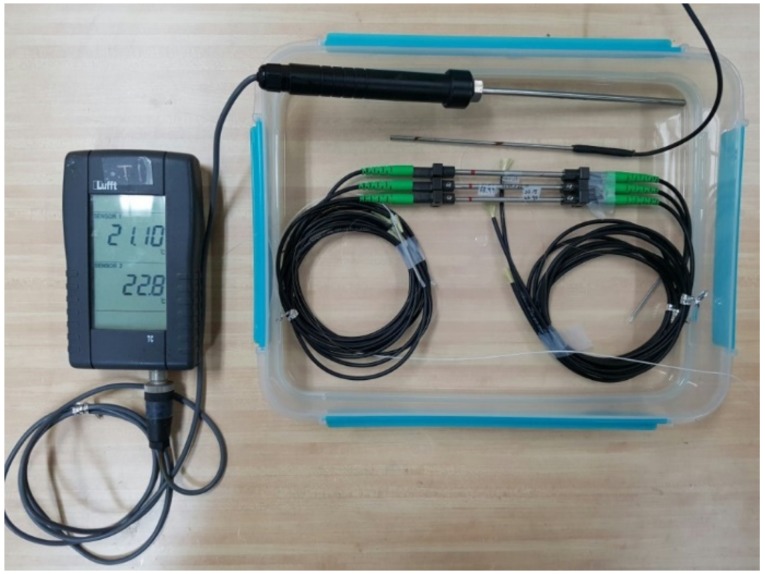
Submergence test of the FBG sensors.

Since the FBG sensor is to be installed in the ocean, an experiment to determine the wavelength changes between pre-immersion and after immersion was performed, as shown in Figure 5. The conventional strain sensors and the developed FBG strain sensors were submerged in the same ocean water sample. The experiment was conducted to determine the differences of the wavelength for comparing conventional strain sensors with developed FBG strain sensors in marine environment.

**Figure 6:**
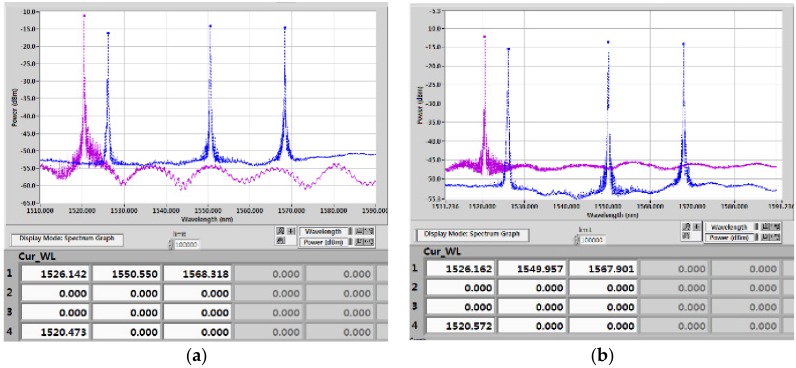
Results of the submergence test of: (**a**) the conventional strain sensors; and (**b**) the FBG strain sensors.

The results of the submergence test for the conventional strain sensors and FBG sensors are shown in Figure 6 and the data are organized in Table 4. Table 4 shows that the conventional strain sensors had an average wavelength difference of −0.1063 nm and submerging the sensor in water can affect the measurement of the sensors. However, the developed sensor had an average wavelength difference of −0.0006 nm, which was not affected by submergence in water. Therefore, this result indicates that the developed sensor and cable can make precise measurements in marine environments.

#### 2.1.6. Calibration of the FBG Sensors

**Figure 7:**
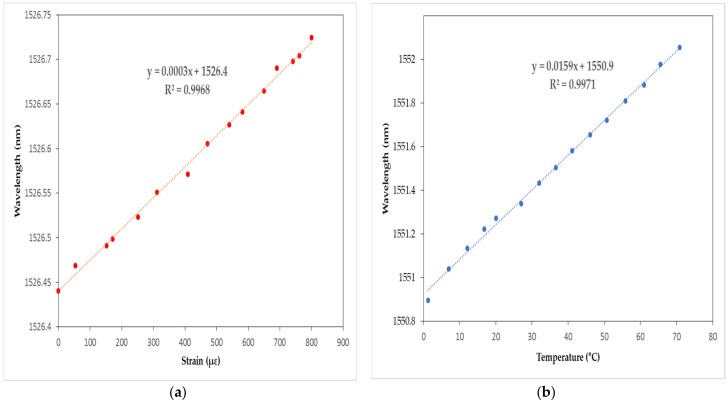
Calibration results of FBG: (**a**) strain sensor; and (**b**) temperature sensor.

A calibration of FBG sensors were applied in a bare FBG in this study. The bare FBG sensor and encapsulated sensor were affixed to the opposite end of a steel coupon. Epoxy resin was used as the bonding material for the bare FBG; cyanoacrylate was used as the adhesive agent for the FBG strain gauge. Then, the steel coupon was put into a universal testing machine to determine the variations of Δλ_F_ and ε under the applied conditions. Figure 7a presents the linear relationship between Δλ_F_ and ε with a strain sensitivity of 0.3 pm/με. Excluding any possible effects of strain and temperature, the relationship can be stated as

(2) $$\Delta \lambda_{F}\;=\;0.0003x\;+\;1526.4$$

A mercury thermometer with 0.05 °C accuracy and three FBG temperature sensors with central wavelengths were used. A standard bath method was selected to note the temperature sensitivity coefficient of the FBG strain sensors. Each FBG sensor was located separately in a testing tub with temperatures of 10, 15, and 20 °C with rises of 1 °C. The linearity constant between Δλ_F_ and ΔT was observed to be 0.99 with a temperature sensitivity of 1.6 pm/°C, presented in Figure 7b and given by

(3) $$\Delta \lambda_{F}\;=\;0.0159x\;+\;1550.9$$

### 2.2. Gateway

The gateway consists of a sensing interrogator module and wireless module to gather the data and LTE module to transmit the data from the sensors installed in the port. A sm 125–500 module by Micron Optics was used for the platform that has a four optical channel and displays the full spectrum in the wavelength range of 1510 to 1590 nm. The scan frequency is 2 Hz with a dynamic range of 50 dB with FBG sensor capacity of 60 to 120. The interrogator module gathers data from the FBG sensors via a wireless module and outputs wavelength data to the web server via the LTE module.

The LTE module, an RCU890L LTE modem from Woojin Networks, was installed to transmit the data to the web servers for analysis. The modem is a mobile communication terminal device which can transmit and control data in real time for the platform. Therefore, the modem is a connection between the FBG sensors and web server, the specifications of which are shown in Table 5.

### 2.3. Amazon Web Services

The cloud computing-based pier type harbor structure stability evaluation platform needs a server to efficiently analyze the data from the FBG sensors and visualize the analysis for the platform. Amazon Web Services (AWS) was used as the server, as it is a commercial cloud computing platform that is widely utilized in many applications. Since developing a platform would require a lot of time and money to minimize the errors and get certified, AWS was used as the platform because it is a certified open platform that offers open source libraries. AWS provides rich support for management of the computation server, which assists in monitoring the structure efficiently. In addition, AWS provides a database to save the data and analyze the platform while it does not require the installation of a separate database [19,20,21].

Among Amazon-supported application programming interfaces (APIs) for hypertext preprocessor (PHP), EC2 (elastic compute cloud) was used for the cloud computing-based port structure stability evaluation platform. EC2 provides support for the dynamic instantiation and configuration of the virtual machine instance, which is suitable for the platform. The platform uses a T2 medium as an extensible instance that are listed in Table 6.

## 3. Development of a Cloud Computing-Based Pier Type Port Structure Stability Evaluation Platform

**Figure 8:**
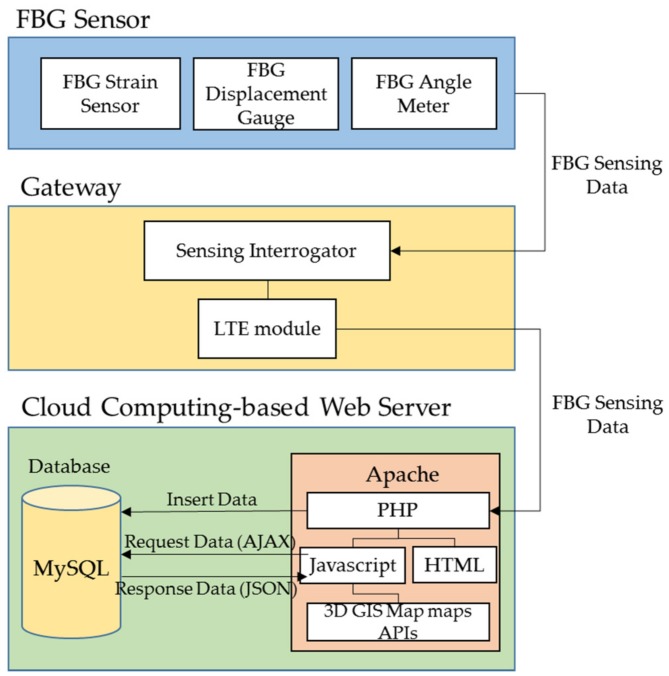
Configuration diagram of the cloud computing-based pier type port structure stability evaluation platform.

The cloud computing-based pier type harbor structure stability evaluation platform has primarily been divided (Figure 8) into three parts: FBG sensors, gateway, and cloud computing-based web server. The FBG sensors measure data needed to determine the safety evaluation index (SEI). The gateway connects FBG sensors with the web server using an LTE module. The web server is the main part of the platform and analyzes the data to determine the SEI and visualize the data. The server was designed with PHP, a web programming language, while MySQL was used as the database for the platform.

**Figure 9:**
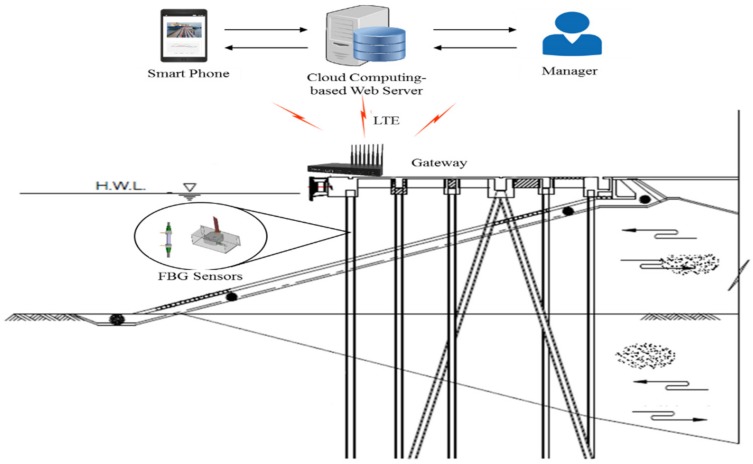
The cloud computing-based pier type port structure stability evaluation platform configuration diagram.

Furthermore, a system diagram of the cloud computing-based pier type harbor structure stability evaluation platform is shown in Figure 9. For effective measurements, the FBG sensors were installed at the most appropriate place on the structure surfaces. These sensors collected data, which are sent to the web server through a gateway. The main server analyzed the collected data to evaluate the SEI based on “port and fishing port design standards.” The managers, inspectors, and users with specified access to the monitoring data can continuously monitor the structure with anytime and anywhere features using smart devices. Another feature of the main server is that it automatically sends an alarm alert to managers and other related personnel via smartphone-based SMS and popup alarms whenever the SEI values are low. Thus, the proposed system enhances the port structure safety.

### 3.1. Location and Number of the FBG Sensors

Important tasks in designing the FBG network include the sensor installation at the most appropriate location of the structure and determination of the number of sensors such that maximum information can be collected with a minimum number of sensors. According to the “port and fishing port design standards” for static structure and seismic analyses, an important tool is SAP, which analyzes the behavior of the structure under different loadings and performs safety evaluation of the structure. This analysis is helpful for determination of the most appropriate location and number of FBG sensors [3,22]. The analysis revealed the weak parts of the harbor structure under different loadings and moments. The number and the location of the FBG sensors were decided and sensors were installed at the monitoring of the core parts of structure.

### 3.2. Cloud Computing-Based Web Server

The server is used to analyze the data to determine the safety evaluation index for each component of the structure to which sensors are attached. Then, the server is able to visualize the analysis to allow the manager to monitor the structure anytime and anywhere as well as take action depending on the results of the index. Furthermore, the server saves the data into the database which can be reviewed at later stages. However, the most important role of the web server is to monitor the structure based on the SEI of core components of the structure calculated using structural analysis.

#### 3.2.1. Safety Evaluation Index for the FBG Strain Sensors

The safety evaluation index for the strain sensor is determined by measuring the strain of the pile in the structure. According to the “Port and Fishing Port Design Standards” for a pier type harbor structure, the safety evaluation of the external force of each member of the structure is reviewed by applying an allowable stress design method or ultimate strength method, depending on the characteristics of the structure. For pier type structures, the ratio of the actual moment to the design moment of each upper structural member and the lower structural reinforced concrete is calculated according to the ultimate strength method. The ratio of the actual stress to the allowable stress of each of the lower structural members is calculated according to the allowable stress design method to define the conditions of the members, as Equation (4) demonstrates. The ratio is the safety factor (S.F.) of the pile evaluated. In addition, the effects of earthquakes according to the method of the standards are considered in the ratio for evaluating the safety evaluation index.

(4) $$S.F.\;=\;\frac{\mathrm{Allowable}\;\mathrm{Stress}}{\mathrm{Actual}\;\mathrm{Stress}}$$

The condition varies from the S.F. which determines the SEI of the pile. Table 7 shows that the index is set to “A” when the condition is above 1.0, indicating that the design criteria are satisfied. Further, conditions of less than 1.0, less than 0.9, and less than 0.75 were set as “C”, “D”, and “E”, respectively, representing that the design criteria are unsatisfied.

#### 3.2.2. Thresholds for the Strain Data

The threshold limits for strain data are basic requirements to calculate the SEI to monitor a port structure. After locating the sensor attachment to the pile using SAP, structural calculations such as reviewing the member forces and composite stress for the piles are required to calculate the threshold values. For piles in the port structure, the stresses are a combination of compressive stress and bending stress, as described in Equation (5). Both the actual and allowable stresses for compressive stress can be calculated from the structural calculation.

(5) $$S.F.\;=\;\frac{\mathrm{Allowable}\;\mathrm{Stress}}{\mathrm{Actual}\;\mathrm{Stress}}\;=\;\frac{f_{\mathrm{ba}}}{f_{b}}\;+\;\frac{f_{\mathrm{ca}}}{f_{c}}$$

where f_b_ and f_c_ are the actual stresses and f_ba_ and f_ca_ are allowable stresses for bending and compressive stresses, respectively. The actual bending stress, f_b_, is a combination of the bending stress from an overburden and dead load, f_b1_, and external load, f_b2_, for the pile as follows.

(6) $$f_{b}{\;=\;f}_{b1}{\;+\;f}_{b2}$$

where f_b1_ is calculated by the structural calculation and the bending stress originating from an external load is calculated from the measured strain sensor as follows:

(7) $${\;f}_{b2}\;=\;\varepsilon \cdot E$$

where ε is the strain value measured from the sensors. However, if the strain value is assigned unknowingly and the S.F. is assigned a minimum value from the index, the thresholds of the strain data for each safety index can be calculated from inserting Equation (6) into Equation (5) to produce the following equations.

(8) $$\frac{f_{\mathrm{ba}}}{f_{b}}\;+\;\frac{f_{\mathrm{ca}}}{f_{c}}\;=\;S.F.\Rightarrow \frac{f_{\mathrm{ba}}}{f_{b1}{+f}_{b2}}\;=\;S.F.\;-\;\frac{f_{\mathrm{ca}}}{f_{c}}$$

(9) $$\Rightarrow \frac{f_{b1}\;+\;f_{b2}}{f_{\mathrm{ba}}}\left[S.F.\;-\;\frac{f_{\mathrm{ca}}}{f_{c}}\right]\;=\;1\Rightarrow \frac{1}{S.F.}\;-\;\frac{f_{c}}{f_{\mathrm{ca}}}\;=\;\frac{f_{b1}\;+\;f_{b2}}{f_{\mathrm{ba}}}$$

(10) $$\Rightarrow \left(\frac{1}{S.F.}\;-\;\frac{f_{c}}{f_{\mathrm{ca}}}\right)f_{\mathrm{ba}}{\;=\;f}_{b1}{\;+\;f}_{b2}\Rightarrow \left(\frac{1}{S.F.}\;-\;\frac{f_{c}}{f_{\mathrm{ca}}}\right)f_{\mathrm{ba}}\;-\;f_{b1}\;=\;f_{b2}$$

(11) $$∴\;\varepsilon \;=\;\left[\left(\frac{1}{S.F.}\;-\;\frac{f_{c}}{f_{\mathrm{ca}}}\right)f_{\mathrm{ba}}{\;-\;f}_{b1}\right]/E$$

Since the index has four grades from the standards, three thresholds are determined by the minimum values from each grade. Safety factor values of 1.0, 0.9, and 0.75 are used to determine the 1st, 2nd, and 3rd thresholds, respectively, as shown in the following equations.

(12) $$1\mathrm{st}\;\mathrm{Threshold}\Rightarrow \;\varepsilon \;=\;\left[\left(\frac{1}{1.0}\;-\;\frac{f_{c}}{f_{\mathrm{ca}}}\right)f_{\mathrm{ba}}\;{-\;f}_{b1}\right]/E$$

(13) $$2\mathrm{nd}\;\mathrm{Threshold}\Rightarrow \;\varepsilon \;=\;\left[\left(\frac{1}{0.9}\;-\;\frac{f_{c}}{f_{\mathrm{ca}}}\right)f_{\mathrm{ba}}\;{-\;f}_{b1}\right]/E$$

(14) $$3\mathrm{rd}\;\mathrm{Threshold}\Rightarrow \varepsilon \;=\;\left[\left(\frac{1}{0.75}\;-\;\frac{f_{c}}{f_{\mathrm{ca}}}\right)f_{\mathrm{ba}\;}{-\;f}_{b1}\right]/E$$

Therefore, the safety evaluation index is “A” until the strain data exceed the 1st threshold. When the data exceed the 1st, 2nd, and 3rd thresholds, the safety evaluation index values are “C”, “D”, and “E”, respectively.

#### 3.2.3. Safety Evaluation Index for the Displacement Data

The FBG displacement gauge is installed between the cap concrete and superstructure to detect subsidence for the pier type port structure. According to the “Port and Fishing Port Design Standards” for a pier type harbor structure, the evaluation standard for the subsidence depends on the maximum settlement, which is based on the investigated state. The SEI is set as “A” when subsidence has not occurred and different index values are set based on the subsidence value, as shown in Table 8.

The FBG displacement gauge measures the settlement for which the thresholds for the displacement data are minimum values of the settlement for each index. Thus, the thresholds for the 1st, 2nd, 3rd, and 4th thresholds for unprogressive subsidence are 5 cm, 8 cm, 12 cm, and 16 cm and the corresponding SEIs are “B”, “C”, “D”, and “E”, respectively. In addition, the 1st, 2nd, 3rd, and 4th thresholds for progressive subsidence are 2 cm, 5 cm, 8 cm, and 12 cm with safety evaluation indices of “B”, “C”, “D”, and “E”, respectively.

#### 3.2.4. Safety Evaluation Index for the Angle Meter Data

An angle meter was installed on the cap concrete of the structure to measure the slope for monitoring the port structure. According to the standards, the evaluation standard is dependent on the maximum angle of slope which is based on the investigated state. In addition, the SEI is set as “A” when the slope has not occurred and different index values are set depending on the slope angle, as shown in Table 9.

The FBG angle meter measures the angle of the slope directly and the thresholds for the angle meter data are the minimum angles of the slope for each index. Thus, the 1st, 2nd, 3rd, and 4th thresholds for unprogressive slope are 1.8°, 2.7°, 3.6°, and 5.4° with safety evaluation indices of “B”, “C”, “D”, and “E”, respectively. The 1st, 2nd, 3rd, and 4th thresholds for progressive slope are 0.9°, 1.8°, 2.7°, and 3.6° with safety evaluation indices of “B”, “C”, “D”, and “E”, respectively.

### 3.3. Total Safety Evaluation Index

According to the “Port and Fishing Port Design Standards” for a pier type harbor structure, the total SEI of the structure is the lowest index of the SEI obtained from the sensors. Thus, the web server will automatically select the lowest index from the sensors for the platform [3,4].

### 3.4. Sending Alerts

A grade of “A” in the index indicates the best condition with no defects. Furthermore, when the total SEI is not “A”, the structure is considered as damaged or dangerous and the manager or people who are involved in the structure need to know the condition of the structure. Therefore, the web server is designed to alert the manager and people who are involved in the structure via an SMS message through a smart phone and pop-up message through a web page when the data are over the thresholds. The AWS provides the application for the alert system in an open library that is used in the platform.

## 4. Experimental Testing

Experimental efforts have focused on practical implementation subjects. The Maryang harbor in Korea was evaluated to test the durability of the cloud computing-based pier type port structure stability evaluation platform. The harbor is a pier-type port structure located in Marayng-Meyon, Korea, which was constructed in 1999. Complete installation of platform ended in August 2017. The installation was helpful to evaluate the safety state of structure. Entire installation was comprised of FBG sensors, gateway, and cloud computing-based web server.

### 4.1. Installation

**Figure 10:**
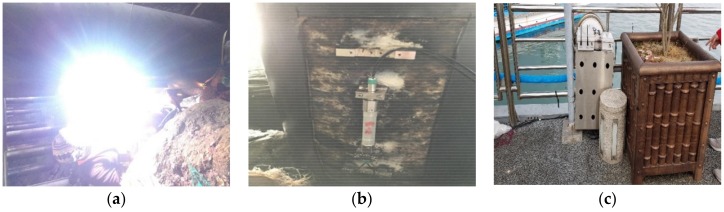
(**a**) Underwater welding of the FBG strain sensor; (**b**) installed FBG strain sensors on the pile; and (**c**) gateway in the secured case installed on the port structure.

The location and number of the FBG sensors were defined by structural analysis using SAP. From the structural analysis of the harbor, eight piles and one concrete cap were identified as the weak part bearing various loadings and moments which required the monitoring. Therefore, sixteen FBG strain sensors were installed for evaluating the piles; two FBG strain sensors for each pile installed at mid-point location and at end of pile. Two FBG displacement gauges and one FBG angle meter were selected for a concrete cap; two FBG displacement gauges were attached in horizontal and vertical configurations in subject to monitor. Figure 10a,b shows the installed sensors in the harbor including underwater welding and sensors. In addition, the gateway consists of a data logger with LTE and wireless module, which is installed in a secured case to protect it from theft, as shown in Figure 10c.

### 4.2. Data Transmission

**Figure 11:**
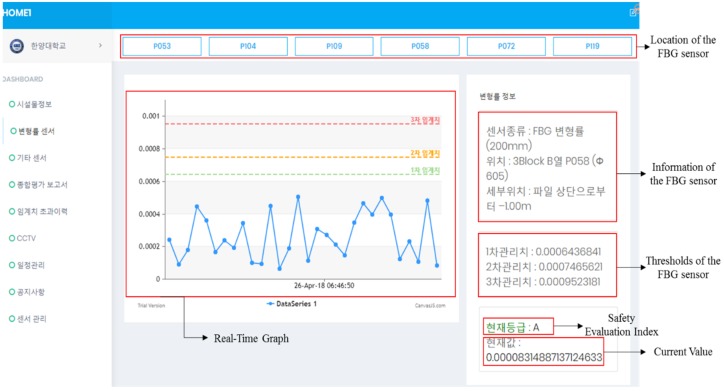
A screenshot of the web server that displays the measured data.

After installing the platform in the Maryang harbor, a test was focused on the data transmission of the gateway. The data were well stored and analyzed in the web server of the platform, as shown in Figure 11. The figure shows a screenshot of the web server that displays measured data from the FBG sensors, which the web server received via the LTE module from the gateway. By clicking the location of the sensor, its data will be visualized through real-time graph along with the information and thresholds of the sensor. Based on the values from the data, the safety evaluation index of the sensor will be shown on the bottom of the screen.

### 4.3. Thresholds for the FBG Sensors

The thresholds for each of the FBG sensors were calculated for input into the web server. Examples of the calculated thresholds are shown in Table 10 along with their locations. In addition, the thresholds for the FBG displacement gauge and FBG angle meter were assigned according to the SEI of the sensors from the “Port and Fishing Port Design Standards” mentioned above. However, these thresholds are key parts to monitor the analyzed core parts of the structure which define the safety evaluation index through the web server.

### 4.4. Cloud Computing-Based Web Server

**Figure 12:**
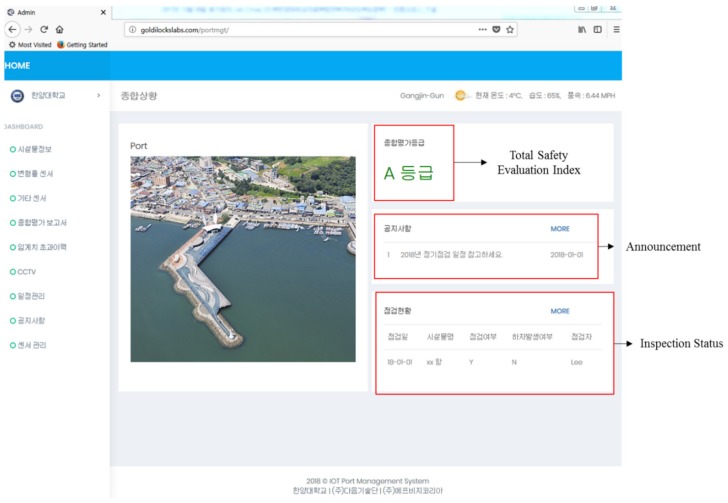
Main page of the web server.

The web server for the Maryang harbor was enabled after connecting the data logger and storing information, including the specifications of the harbor and thresholds for the installed sensors. Figure 12 shows the web server used to monitor the Maryang harbor, where the server address is http://goldilockslabs.com/portmgt/. Furthermore, the total safety index of the structure is displayed in the top right corner of the main page of the server where other monitoring data can be achieved by selecting options in the dashboard located on the left side of the page. Therefore, managers and people who are involved in the structure can monitor the structure anytime and anywhere by accessing the web server.

## 5. Results

**Figure 13:**
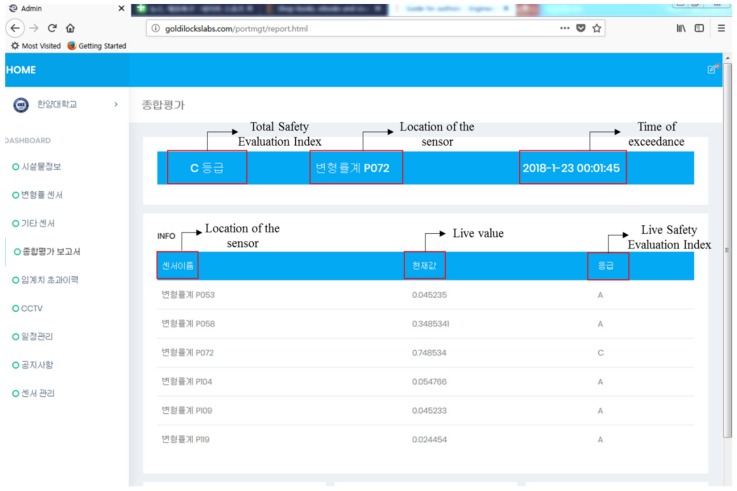
Page for monitoring the Marayng Harbor.

The experiments were focused on implementing the platform in a real pier type harbor to demonstrate a successful test. The designed FBG sensors and cables functioned in the ocean and data was transmitted to the data logger. In addition, the data from the sensors were saved in the server of the platform using the LTE module as designed such that web server can analyze it. After inputting the information and thresholds for the harbor, the server was able to monitor the harbor structure, as shown in Figure 13. The figure is a screenshot of the monitoring the structure, which contains a section of the total safety evaluation index, including the index, sensor name, and time on the top. In addition, the page shows a list of sensors along with the SEI, which can monitor all of the FBG sensors at the same time.

## 6. Conclusions

In this paper, the development of a cloud computing-based pier type port structure stability evaluation platform is presented. Experiments making use of the platform were conducted, which demonstrated the good performance of the platform and the convenience of monitoring the harbor structure. Several achievements of the platform were accomplished, including the following: (1) the cloud computing platform is well suited to efficiently monitor harbor structures especially pier type structures; (2) the harbor structures can be monitored anytime and anywhere using cloud computing technology; (3) the platform used Amazon Web Services as a certified web server; and (4) the platform is also easily extendable such that additional FBG sensors and applications can be installed or additional structures can be monitored.

Future work will involve further testing of the platform. In this paper, the experiment focused on implementing the platform to a real pier type harbor structure, where more tests are necessary to ensure data accuracy for long time periods. In addition, more applications for the platform to monitor the structure can be added such as calculating the berthing force to prevent damaging the structure by installing an FBG accelerometer. This will be studied in the future. If the potential problems that arise from experiments or applications that are needed to monitor the structures are compensated for, the utilization of this approach is expected to grow.

## Figures and Tables

**Table 1:** Specifications of the FBG strain sensor.

Specifications	Values
Measurement Range	±2000 με
Strain Sensitivity	≥1 pm/με
Gage Length	200 mm
Operating Temperature Range	−20 to 80 °C
Dimension	270 mm × 48 mm × 13 mm
Weight	50 g
Cable Type	3 mm lead cable
Fastening Type	Anchor bolt or bolt
Peak Reflectivity	>70%
Center Wave Length Range	1511 nm–1589 nm

**Table 2:** Specifications of the FBG displacement gauge.

Specifications	Values
Measurement Range	≤30 mm
Sensitivity	≥100 pm/mm
Operating Temperature Range	−20 to 80 °C
Dimension	137 mm × 50 mm × 23 mm
Weight	250 g
Cable Type	3 mm lead cable
Fastening Type	Anchor bolt
Peak Reflectivity	>70%
Center Wave Length Range	1511 nm–1589 nm

**Table 3:** Specifications of the FBG angle meter.

Specifications	Values
Measurement Range	6 degrees (−3 to 3)
Sensitivity	≥450 pm/°
Operating Temperature Range	−20 to 80 °C
Dimension	87 mm × 41 mm × 93 mm
Weight	≤1.2 kg
Cable Type	3 mm lead cable
Fastening Type	Anchor bolt
Peak Reflectivity	>70%
Center Wave Length Range	1511 nm–1589 nm

**Table 4:** Results of the submergence test.

Sensors	Conventional Strain Sensor (Unit: nm)			FBG Strain Sensor (Unit: nm)		
	Wavelength before Immersion	Wavelength after Immersion	Change in Wavelength	Wavelength before Immersion	Wavelength after Immersion	Change in Wavelength
1	1526.248	1526.142	−0.106	1526.162	1526.162	0
2	1550.663	1550.550	−0.113	1549.957	1549.957	0
3	1568.418	1568.318	−0.1	1567.903	1567.901	−0.002
	Average = −0.106333333			Average = −0.000666667		

**Table 5:** Specifications of the LTE module.

Performance Properties	RCU890L LTE Module
Communication Method	LG U + LTE B5/B7 FDD Cat.4
Interface	DB9 RS-232, RJ-45 Ethernet, GPIO
Data Speed	150 Mbps DL/50 Mbps UL
Band	LTE FDD 850 MHz(B5)/2.6 GHz(B7)
Input Voltage	4.5 V to 5.5 V

**Table 6:** Specifications of the instance for AWS [19,20,21].

Type of Instance	vCPUs	Memory (GiB)	Storage (GB)	CPU Credits/h	Clock Speed (GHz)	Networking Performance
t2.medium	2	4	EBS Only	24	Maximum 3.3	Low–Medium

**Table 7:** Total safety evaluation index (SEI) for the cloud computing-based pier type port structure stability evaluation platform.

Condition	Rating	Safety Evaluation Index (SEI)
S.F. ≥ 1.0	5	A
0.9 ≤ S.F. < 1.0	3	C
0.75 ≤ S.F. < 0.9	2	D
S.F < 0.75	1	E

**Table 8:** Safety Evaluation Index (SEI) for the FBG displacement sensor.

Safety Evaluation Index Index	Rating	Maximum Settlement Range	
		Unprogressive	Progressive
A	5	Under 5 cm	Under 2 cm
B	4	5 cm–8 cm	2 cm–5 cm
C	3	8 cm–12 cm	5 cm–8 cm
D	2	12 cm–16 cm	8 cm–12 cm
E	1	>16 cm	>12 cm

**Table 9:** Safety Evaluation Index (SEI) for the FBG angle meter.

Safety Evaluation Index	Rating	Maximum Slope Range	
		Unprogressive	Progressive
A	5	Under 1.8°	Under 0.9°
B	4	1.8°–2.7°	0.9°–1.8°
C	3	2.7°–3.6°	1.8°–2.7°
D	2	3.6°–5.4°	2.7°–3.6°
E	1	>5.4°	>3.6°

**Table 10:** Calculated thresholds for the FBG sensors.

	Location of the FBG Sensors					
	(P109, 1.00 m from the Top)	(P109, 3.95 m from the Top)	(P058, 1.00 m from the Top)	(P058, 5.10 m from the Top)	(P119, −1.00 m from the Top)	(P072, 0.75 m from the Top)
1st Threshold	0.000643684	0.00065965	0.000477608	0.000476754	0.00063922	0.00057976
2nd Threshold	0.000746562	0.000762528	0.000580487	0.000579632	0.000742098	0.000682638
3rd Threshold	0.000952318	0.000968284	0.000786243	0.000785388	0.000947855	0.000888394
